# Intramedullary spinal cord neurocysticercosis presenting as Brown-Séquard syndrome

**DOI:** 10.1186/s12883-014-0245-5

**Published:** 2015-01-16

**Authors:** Elda M Salazar Noguera, Rita Pineda Sic, Fernando Escoto Solis

**Affiliations:** Department of Internal Medicine, Hospital General San Juan de Dios, Guatemala, Guatemala; Department of General Surgery, Hospital General San Juan de Dios, Guatemala, Guatemala

**Keywords:** Intramedullary, Neurocysticercosis, Spinal cord

## Abstract

**Background:**

Cysticercosis is a parasitic disease caused by the larval stage of *Taenia Solium*. Involvement of the central nervous system by this tapeworm is endemic in developing countries. However, isolated spinal involvement by *Taenia Solium* is uncommon and having clinical presentation of Brown-Séquard syndrome is even rarer.

**Case presentation:**

A 43-year-old male who came to the emergency department with clinical presentation of complete Brown-Séquard syndrome. Computed tomography scan of the brain was normal. Magnetic resonance imaging of the thoracic spine revealed an intramedullary mass of the spinal cord at C-7/T-l level. Patient underwent surgery that revealed a cystic lesion and was resected. Histopathological report confirmed the diagnosis of neurocysticercosis. Postoperatively, oral steroid therapy and a four week course of albendazol were administered.

**Conclusions:**

Intramedullary neurcysticercosis represents a diagnostic challenge and should be considered in intramedullary lesions in settings where *Taenia solium* is endemic. Clinical, pathophysiological and diagnostic aspects of spinal cord intramedullary neurocysticercosis are discussed.

## Background

Cysticercosis is caused by the encysted larvae stage of the pork tapeworm *Taenia Solium* [1]. Cysticercosis is the most common parasitic disease of the central nervous system (CNS) in developing countries, but spinal cysticercosis is rare and presenting itself as Brown-Séquard syndrome is even rarer [2]. Only fifty six cases have been reported until 2013 of intramedullary neurocysticercosis (NCC) [2]. Clinical syndromes related to this parasite are divided into neurcysticercosis and extra-neural cysticercosis. Neurocysticercosis in turn is divided into parenchymal and extra parenchymal forms. Extra parenchymal forms include intraventricular, subarachnoid, intraocular and spinal disease [3]. The spinal type is rare, accounts for only 1.0-5.8% of cases, and isolated spinal NCC without intracranial involvement is extremely rare [3-5].

Here, we report a case of isolated intramedullary NCC exhibiting clinical features of Brown-Séquard syndrome.

## Case presentation

A previously healthy 43-year-old man presented to the emergency room of the San Juan de Dios General Hospital Guatemala City, Guatemala with a history of twenty days insidious onset of gradual progressive numbness of the left lower extremity and weakness of the right lower extremity. He had two episodes of urinary incontinence and at time of evaluation he could not walk without assistance. There was no history of fever, weight loss, night sweats or trauma.

**Figure 1:**
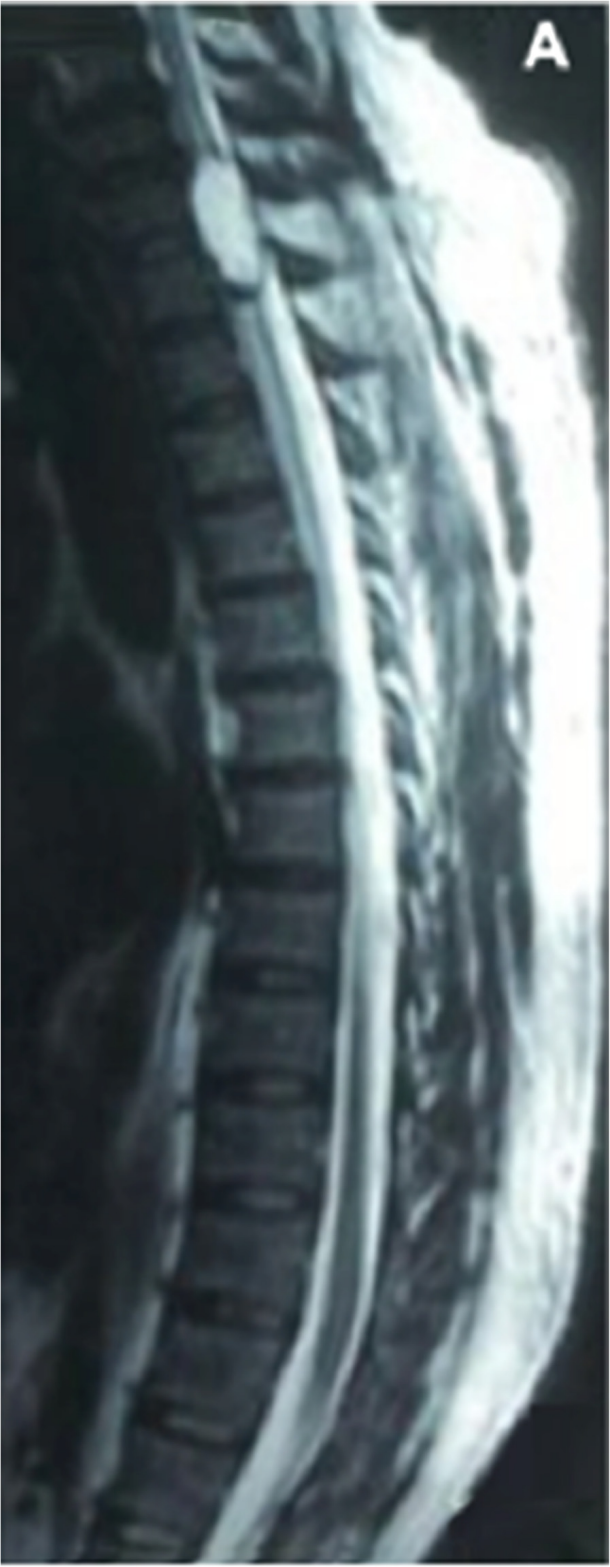
**Thoracic MRI.** Sagital T2 MR image of the thoracic region demonstrating round intramedullary mass at C7 to T1 level meassuring 33 × 12 mm.

On physical examination he was afebrile with stable vital signs. Neurological examination of mental status, cranial nerves and upper extremities were normal. Examination of the right lower extremity revealed: hyperreflexia, 2/5 strength of all muscle groups. Sensation was decreased to mild palpation, vibration and position. Motor examination of the left lower extremity showed normal strength. Sensation was decreased to noxious stimulation from T4 dermatome to the plantar surface of the left foot. Babinski sign was present on the right side. A non-contrasted computer tomography (CT) scan of the brain showed no abnormalities. Magnetic resonance imaging (MRI) of the thoracic spine revealed a contrast-enhancing intramedullary mass extending from C7 to T1 (Figure 1).

**Figure 2:**
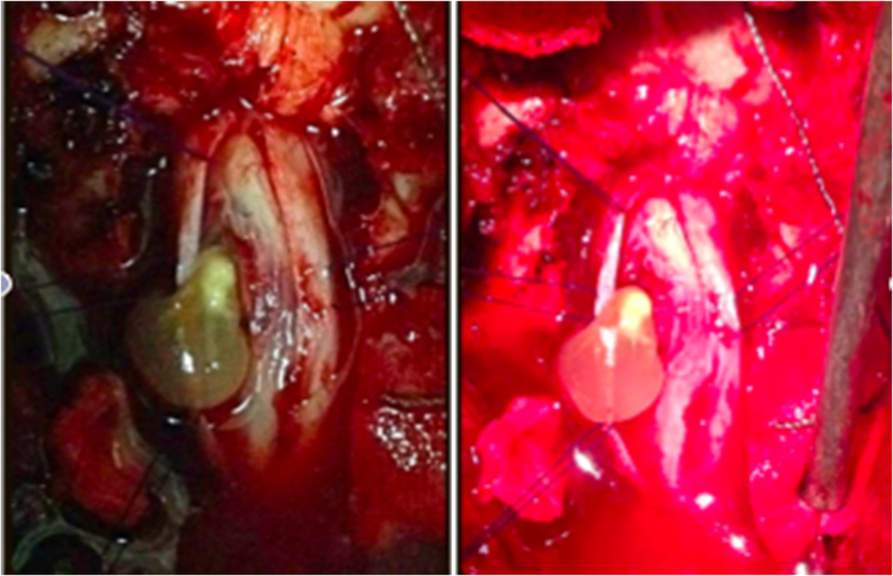
**Surgical excision.** Mielotomy showing the intramedullary cyst, after excision it was possible to see the cyst in its vesicular stage and the larva inside with a marginal projecting nodule (scolex) surrounded by clear cyst fluid within a capsule.

The initial differential diagnosis included astrocytoma and ependymoma. The patient was admitted to the internal medicine ward and underwent neurosurgical procedure. Laminectomy of C-6, C-7 and T-1 was performed and after dural opening at the posterior midline, the spinal cord was visualized swollen and containing a cyst localized to the left lateral aspect of the cord, with cord displaced to the right. Cyst resection was performed (Figure 2).

**Figure 3:**
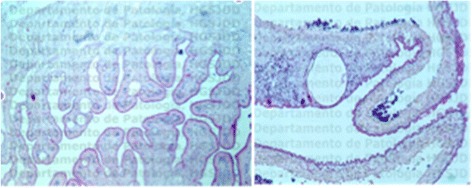
**Histological specimen.** Cross-section of cysticerci stained with H&E.

Histopathological findings showed chronic inflammation associated with the cysticercous cyst (Figure 3).

After surgery, the strength of his right lower limb recovered to grade 3/5. Postoperatively, oral steroid therapy and anticysticercal agent (albendazol) were administred for four weeks. Patient was discharged to physical rehabilitation therapy. After one month, steroid therapy was tapered and after the 5-month-follow-up period the patient recovered normal muscle function and was able to ambulate without assistance.

## Discussion

Cysticercosis is endemic in low-income and lower-middle income conuntries, mainly affecting Africa, Latin America and Asia [2,3]. Cysticerci spinal cord involvement is rare and varies from 1% to 5% of all cases of neurocysticercosis, even in endemic areas [3]. Spinal forms have been identified in the vertebral, extradural, intradural and intramedullary region. Intramedullary cysticercosis is very uncommon and only fifty-six cases have been reported so far [2].

In addition, many cases with intracranial subarachnoid cysticercosis also have spinal lesions. Migration of the cysticercus through the ventricular-ependymal pathway and hematogenous dissemination has been identified to be the possible pathogenic mechanism [6].

Spinal cysticerci are usually located in the subarachnoid space where they can cause inflammatory and demyelinating changes in the peripheral nerve roots. Patients typically present radicular pain, paresthesia and /or sphincter disturbances. Neurological deficits vary with the location of the lesion and may not be distinguishable from other spinal cord lesion on clinical grounds alone [6,7].

Most patients with cysticercosis have no specific diagnostic findings on a complete blood count and liver function tests. Peripheral eosinophilia is usually absent and stool examination is insensitive.

Diagnosis of neurocysticercosis begins with CT imaging of the brain and serology with enzyme-linked immunoelectrotransfer blot assay (EITB). If the CT scan is inconclusive subsequent MRI is appropriate [8-10].

Brown-Séquard syndrome was described by neurologist Charles-Edouard Brown Séquard to describe the clinical syndrome accompanying hemi section of the spinal cord. The classic syndrome involves crossed findings, with hemiplegia, hyperrreflexia and loss to light touch and proprioception affecting the ipsilateral side and sensory defects of painful touch and temperature affecting the contralateral side. This asymmetrical presentation results from the crossing of the neural fiber tracts at different levels within the CNS [2].

Our patient presented with complete Brown-Séquard syndrome, rarely seen in clinical practice. This is the third case report in the literature of intramedullary NCC presenting as Brown-Séquard syndrome [2,11].

Surgical results have not been conclusive in intramedullary cysticercosis and some reports indicate success with medical treatment alone. Mohanty et al. reported only a 75% satisfactory outcome after surgery and cysticidal treatment. Sharma et al. reported that 60% had improvement after surgery, 25% did not and 15% died [12]. However, in cases were patients have neurological deficit surgery should be considered as the initial treatment of choice due to risk of progressive spinal cord compression. In contrast, the potential advantages of medical therapy alone include avoiding surgery in high risk patients and treatment of surgically unreachable cysticercus [13-16]. Postoperatively, anticysticercal drugs should be instituted in all cases.

## Conclusions

Intramedullary neurocysticercosis represents a diagnostic challenge and should be considered for intramedullary lesions. Though various therapeutic options exist for spinal neurocysticercosis, the rarity of spinal involvement has precluded the evolvement of definite guidelines. Medical treatment alone can be considered in selected cases.

## Consent

The patient gave written informed consent for the publication of the accompanying images and this report. The authors are available for any clarification. The publication was approved by the ethic committee of the San Juan de Dios General Hospital.

## Abbreviations

CT: Computed tomography
MRI: Magnetic resonance imaging
H&E: Hematoxylin and eosin
NCC: Neurocysticercosisl
CNS: Central nervous system
